# Rapid Differentiation between Livestock-Associated and Livestock-Independent *Staphylococcus aureus* CC398 Clades

**DOI:** 10.1371/journal.pone.0079645

**Published:** 2013-11-14

**Authors:** Marc Stegger, Cindy M. Liu, Jesper Larsen, Katerina Soldanova, Maliha Aziz, Tania Contente-Cuomo, Andreas Petersen, Stien Vandendriessche, Judy N. Jiménez, Caterina Mammina, Alex van Belkum, Saara Salmenlinna, Frederic Laurent, Robert L. Skov, Anders R. Larsen, Paal S. Andersen, Lance B. Price

**Affiliations:** 1 Microbiology and Infection Control, Statens Serum Institut, Copenhagen, Denmark; 2 Translational Genomics Research Institute, Pathogen Genomics Division, Flagstaff, Arizona, United States of America; 3 Laboratoire de Référence MRSA-Staphylocoques, Hôpital Erasme, Brussels, Belgium; 4 Escuela de Microbiologia, Grupo de Microbiología Molecular, Universidad de Antioquia, Medellín, Colombia; 5 Department of Sciences for Health Promotion “G. D’ Alessandro”, University of Palermo, Palermo, Italy; 6 bioMérieux, Microbiology R&D, La Balme les Grottes, France; 7 Department of Infectious Disease Surveillance and Control, National Institute for Health and Welfare, Helsinki, Finland; 8 National Reference Center for Staphylococci, Laboratory of Bacteriology, Hôpital de la Croix Rousse, Inserm U851, Lyon, France; 9 Department of Environmental and Occupational Health, George Washington University, Washington DC, United States of America; National Institutes of Health, United States of America

## Abstract

*Staphylococcus aureus* clonal complex 398 (CC398) isolates cluster into two distinct phylogenetic clades based on single-nucleotide polymorphisms (SNPs) revealing a basal human clade and a more derived livestock clade. The *scn* and *tet*(M) genes are strongly associated with the human and the livestock clade, respectively, due to loss and acquisition of mobile genetic elements. We present canonical single-nucleotide polymorphism (canSNP) assays that differentiate the two major host-associated *S*. *aureus* CC398 clades and a duplex PCR assay for detection of *scn* and *tet*(M). The canSNP assays correctly placed 88 *S. aureus* CC398 isolates from a reference collection into the human and livestock clades and the duplex PCR assay correctly identified *scn* and *tet*(M). The assays were successfully applied to a geographically diverse collection of 272 human *S. aureus* CC398 isolates. The simple assays described here generate signals comparable to a whole-genome phylogeny for major clade assignment and are easily integrated into *S. aureus* CC398 surveillance programs and epidemiological studies.

## Introduction

Livestock has been considered the primary reservoir of methicillin-resistant *Staphylococcus aureus* (MRSA) clonal complex 398 (CC398); however, there is now strong evidence of a livestock-independent *S. aureus* CC398 clade circulating in humans that predates the livestock clade [1]–[4].

Epidemiological studies have shown that most livestock-associated MRSA CC398 (LA-MRSA CC398) strains colonize and transmit between humans to a lesser degree than other MRSA strains [5], although they are an important cause of infection in persons having direct contact with livestock [6]–[8]. The *scn* gene, encoding a staphylococcal complement inhibitor (SCIN) [9], and other genes in the immune evasion cluster (IEC) are likely to play important roles in evasion of the human innate immune response. IEC is largely absent from *S. aureus* CC398 isolates belonging to the livestock clade, whereas it is widespread among livestock-independent *S. aureus* CC398 isolates [3], which may, at least in part, explain the limited spread of livestock-associated *S. aureus* CC398 isolates in humans. In addition, livestock-associated *S. aureus* CC398 isolates carry a number of resistance determinants, including the staphylococcal cassette chromosome *mec* (SCC*mec*) and the *tet*(M) gene encoding methicillin and tetracycline resistance, respectively [3].

The existence of two major host-associated *S*. *aureus* CC398 clades emphasizes the need for rapid molecular genotyping methods in epidemiological investigations and source tracking of *S*. *aureus* CC398. We describe here two assays for defining the phylogenetic origin of *S*. *aureus* CC398. Using these assays, we were able to determine the sources of *S*. *aureus* CC398 recovered from humans and to demonstrate the existence of several *scn*-positive LA-MRSA CC398 isolates that may be readapting to humans.

## Materials and Methods

### Detection and Characterization of Single-nucleotide Polymorphisms

**Figure 1:**
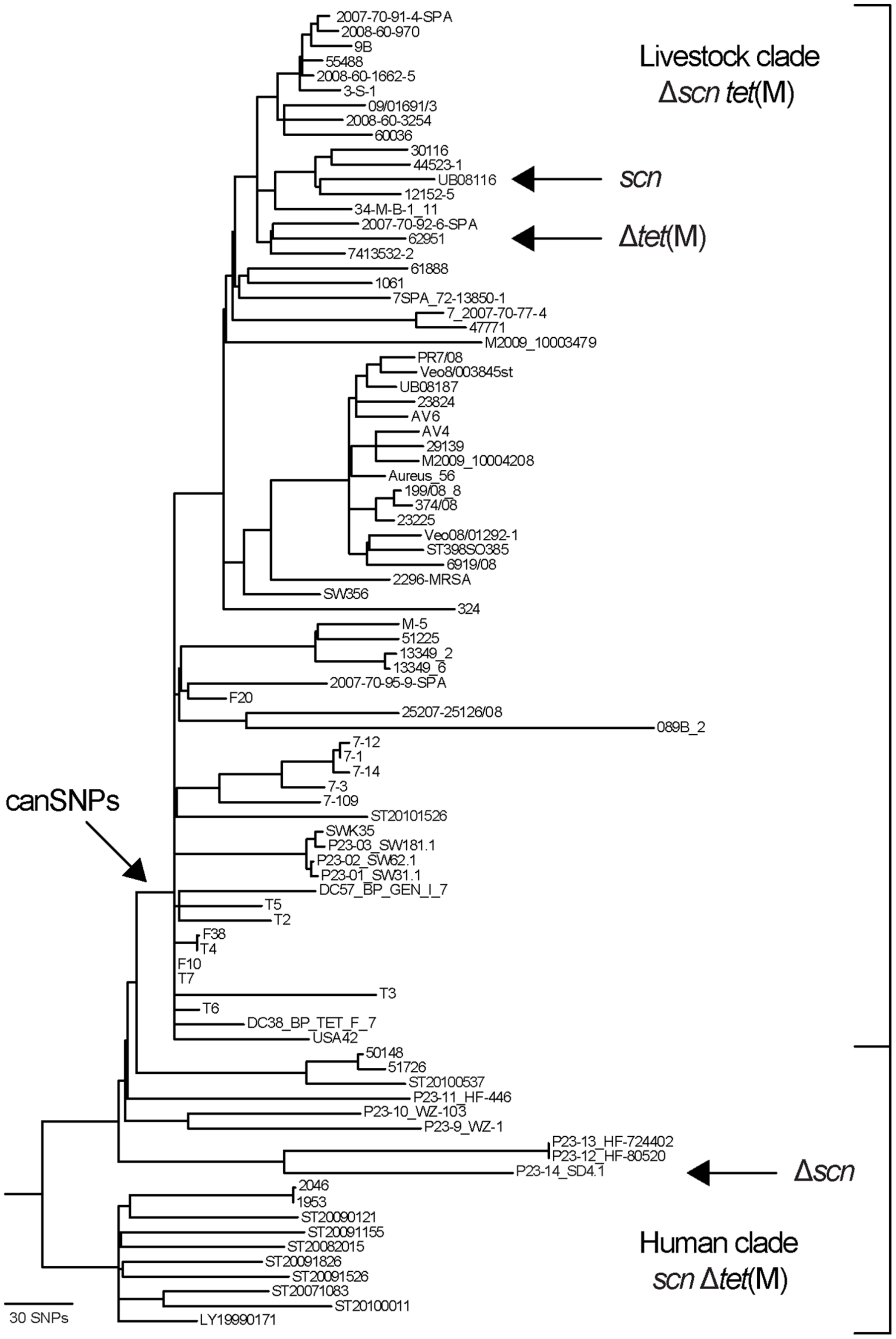
Maximum-parsimony tree of 89 *S. aureus* CC398 isolates based on 4,238 total SNPs, including 1,102 parsimony-informative SNPs. The bracket highlights the ancient human clade and the newly evolved livestock clade. Arrows indicate the position of the branch used to identify canSNPs, and isolates with unique *scn* and *tet*(M) patterns not consistent with the archetypal patterns are highlighted. The figure was adapted from Price *et al*. [3].

The phylogenetic analysis of 89 *S. aureus* CC398 core genomes identified >4,000 single-nucleotide polymorphisms (SNPs) [3]. In the present study, 13 bi-allelic, non-synonymous canonical SNPs (canSNPs) that define the two major host-associated *S. aureus* CC398 clades were identified (Table 1, Figure 1). Of these, three genetically unlinked canSNPs were selected: canSNP_748, canSNP_1002, and canSNP_3737.

**Table 1:** List of bi-allelic, non-synonymous canonical single-nucleotide polymorphisms (canSNPs) that define the two major host-associated *S. aureus* CC398 clades.

canSNP^a^	Genomic position^b^	Codon	
		Humanclade	Livestockclade
15	9,319 (SAPIG0006)	GCC (Ala)	GTC (Val)
237	244,322 (SAPIG0223)	ATG (Met)	ATA (Ile)
476	425,594 (SAPIG0409)	CCA (Pro)	TCA (Thr)
748	551,946 (SAPIG053)	CCA (Pro)	TCA (Thr)
1,002	732,619 (SAPIG0698)	CTA (Leu)	ATA (Ile)
2,167	1,518,366 (SAPIG1434)	GCG (Ala)	GAG (Glu)
2,181	1,524,032 (SAPIG1434)	ATG (Met)	ATA (Ile)
2,761	1,934,659 (SAPIG1823)	CCA (Pro)	CTA (Leu)
3,216	2,287,341 (SAPIG2210)	ATT (Ile)	GTT (Val)
3,399	2,395,959 (SAPIG2317)	CCT (Pro)	CTT (Leu)
3,737	2,597,585 (SAPIG2511)	GGG (Gly)	GAG (Glu)
4,127	2,805,707 (SAPIG2701)	CGC (Arg)	TGC (Cys)
4,130	2,806,556 (SAPIG2701)	ACA (Thr)	TCA (Ser)

a canSNPs used in the study are underlined.

b The genomic position was mapped to the chromosome (genes) of *S*. *aureus* CC398 reference strain SO385 (GenBank accession no. AM990992).

### *S. aureus* CC398 Isolates and DNA Purification

We used a reference collection of 88 *S. aureus* CC398 isolates for which phylogenetic origin and presence of the *scn* and *tet*(M) genes have been previously characterized on the basis of whole genome sequence data [3], and a collection of 272 human *S. aureus* CC398 isolates from ten countries, including Algeria (n = 2), Belgium (n = 5), Colombia (n = 1), Denmark (n = 150), Finland (n = 10), France (n = 94), India (n = 1), Italy (n = 2), Martinique (n = 2), and the Netherlands (n = 5) (Table S1). A subset of 23 isolates has been previously described in other studies [10]–[15].

For the 88 *S. aureus* CC398 reference isolates, DNA was purified using the DNeasy 96 Blood and Tissue Kit (QIAGEN, Valencia, CA, USA) supplemented with lysostaphin in the enzymatic lysis buffer and the Proteinase K-Buffer ATL solution. For the remaining 272 *S. aureus* CC398 isolates, DNA was obtained by incubating the bacteria in distilled water for 10 min at 95°C followed by centrifugation for 5 min at 5,000×*g*.

### canSNP Assays

For each canSNP, 500-bp flanking regions from the chromosome of *S*. *aureus* CC398 reference strain SO385 (GenBank accession no. AM990992) were used to extract the corresponding regions in the 88 whole-genome sequenced *S*. *aureus* CC398 isolates (Short Read Archive accession no. SRS300454-SRS300493, SRS300526-SRS300530, SRS300532, SRS300534-35, SRS300537–SRS300542, SRS300545, SRS300547, SRS300560, SRS300562-63, SRS300565, SRS300567, SRS300569, SRS300571, and SRS300580–SRS300604). The consensus sequence of each flanking region was determined using SeqMan (DNASTAR, Madison, WI, USA), and primers and fluorescently-labelled TaqMan probes were designed using Primer Express version 3.0 (Applied Biosystems, Foster City, CA, USA) (Table 2). Dual-probe real-time PCRs were performed on an ABI 7900 HT Fast Real-Time PCR System (Applied Biosystems) in 10 µL reactions, containing Platinum Quantitative PCR SuperMix-UDG with ROX as a reference dye (Invitrogen Life Technologies, Grand Island, NY, USA), 1 µL of DNA template, 0.6 µM of each primer (Integrated DNA Technologies, San Diego, CA, USA), and 0.2 µM of each probe (Integrated DNA Technologies), with the following settings: 3 min at 50°C, 10 min at 95°C, followed by 40 cycles of 15 s at 95°C, and 1 min at 60°C. All samples were run in duplicate. Allelic discrimination files and multicomponent plots from the ABI 7900HT sequence detection system (Applied Biosystems) were visually inspected to determine the state of each canSNP.

**Table 2:** Primers and TaqMan probes used to identify canonical single-nucleotide polymorphisms (canSNPs) that define the two major host-associated *S*. *aureus* CC398 clades.

canSNPassay	Primers (5′-3′)	Probes (5′-3′)^a^	canSNPclade	Genomic position^b^
748	GGTACTAAGGTATATCCGTGGATTGC	6-FAM-TCTGATTTCATCACCGC	Livestock	551,946 (SAPIG0537)
	ATCAGTTGCGCTAAATCTTCTATTGA	VIC-TCTGATTTCACCACCGC	Human	
1,002	GAAACCAAAGGTAAAACCTAGCAAA	6-FAM-CAACAAGTGTAATATATT	Livestock	732,619 (SAPIG0698)
	AATTAAAGCAATCGGGGTGCT	VIC-CAACAAGTGTAATCTATT	Human	
3,737	TTAYATATTTTTGGTTAACATCTTGCC	6-FAM-TTTAACTTTTGAGTTAGTAGCT	Livestock	2,597,585 (SAPIG2511)
	AAAATAGCTAGTGAAATAATAACTGCGAGT	VIC-TTTAACTTTTGGGTTAGTAGCT	Human	

a TaqMan probes for the specific allele of the two canSNP states (underlined) were fluorescently labeled with either 6-FAM or VIC dye.

b The genomic position was mapped to the chromosome (genes) of *S*. *aureus* CC398 strain SO385 (GenBank accession no. AM990992).

### PCR-based Detection of Adaptive Genetic Markers

The presence of the *scn* and *tet*(M) genes was investigated using a duplex PCR assay and previously published primers [16], [17]; the amplicon sizes were 258 bp and 405 bp, respectively. PCRs were performed using 2 µL of template DNA. A QIAGEN Multiplex PCR Master Mix (QIAGEN) was combined with 0.2 µM of each primer in a 25 µL reaction with the following settings: 15 min at 95°C, followed by 30 cycles of 30 s at 94°C, 90 s at 52.5°C, and 90 s at 72°C, and a final extension of 10 min at 72°C. The *S. aureus* CC398 strains 50148 and 55488 were used as positive controls [3].

## Results

### Validation of the canSNP Assays

The canSNP assays (canSNP_748, canSNP_1002, and canSNP_3737) correctly placed 99% (87/88) of the *S. aureus* CC398 reference isolates into the human clade (n = 19) and the livestock clade (n = 68). For one isolate (F20), two assays correctly placed it in the livestock clade while one assay yielded a negative result for both states (canSNP_1002), despite the presence of conserved primer binding sites as determined by the *de novo* analysis on the whole genome sequence data.

### Validation of the *scn* and *tet*(M) Duplex PCR Assay

The duplex PCR assay correctly identified the *scn* and *tet*(M) genes among the 88 *S. aureus* CC398 reference isolates. The majority (95% [18/19]) of isolates belonging to the human clade carried *scn* and lacked *tet*(M), while a single porcine isolate (P23-14_SD4.1) lacked both genes. Conversely, most (97% [67/69]) of the isolates belonging to the livestock clade carried *tet*(M) and lacked *scn*, while one porcine isolate (62951) lacked both genes and one porcine isolate (UB08116) carried both genes. The sensitivity, specificity, and positive and negative predictive values of *scn* were 0.95, 0.99, 0.95, and 0.99, respectively, for clustering within the human clade, and those of *tet*(M) were 0.99, 1.00, 1.00, and 0.95, respectively, for clustering within the livestock clade.

### Application of Assays

Applying the canSNP assays (canSNP_748, canSNP_1002, and canSNP_3737) on a collection of 272 human *S*. *aureus* CC398 isolates produced congruent results for 97% (265/272) of the *S. aureus* CC398 isolates. For seven isolates, two of the three assays placed them in the human clade while one assay yielded a negative result for both states, including canSNP_1002 (n = 6) and canSNP_3737 (n = 1). Using a two-out-of-three rule for defining *S*. *aureus* CC398 isolates, the canSNP assays placed the *S*. *aureus* CC398 isolates into the human clade (n = 111) and the livestock clade (n = 161). All (100% [111/111]) isolates belonging to the human clade carried *scn* and lacked *tet*(M), whereas most (96% [155/161]) of the isolates belonging to the livestock clade carried *tet*(M) and lacked *scn*. The remaining six isolates belonging to the livestock clade either carried both genes (n = 4) or lacked both genes (n = 2).

## Discussion

We recently showed that the livestock-associated *S*. *aureus* CC398 clade evolved from the basal human clade, and that this human-to-livestock host jump was accompanied by the loss of a bacteriophage (ΦSa3) harboring *scn* and functionally related genes that encode modulators of human innate immunity (IEC) and acquisition of a Tn*916*-like transposon carrying the *tet*(M) gene that confers resistance to tetracycline, which is commonly used in livestock production [3].

In the present study, we developed and validated two rapid molecular genotyping methods for defining the two major host-associated *S*. *aureus* CC398 clades. The three canSNP assays were developed to run optimally under identical conditions and were not affected by the DNA extraction method. Three unrelated SNP positions were included to minimize risk of false clade assignment due to the risk of nucleotide reversal or horizontal gene transfer. Nearly all of the isolates were assigned to the same clade by all three canSNP assays, and only a small subset of isolates yielded a negative result in one of the three assays. To avoid potential misclassifications by the canSNP assays, we suggest instituting a two-out-of-three rule for defining *S*. *aureus* CC398 isolates. As expected, most of the isolates that were assigned to the human clade by use of the canSNP assays carried *scn* and lacked *tet*(M), while the majority of isolates that were placed in the livestock clade carried *tet*(M) and lacked *scn*.

*S*. *aureus* CC398 has been identified in humans with no apparent livestock-associated risk factors in several geographically diverse areas, including the People’s Republic of China [18], Denmark [19], France [20], [21], French Guiana [22], the Caribbean [23], [24], and the United States [23], [25], [26]. Subsequently, whole-genome sequence analysis of *S*. *aureus* CC398 isolates from these geographic areas demonstrated that they belong to the human clade [3], [4]. By use of the assays reported here, we identified the first cases of *S*. *aureus* CC398 belonging to the human clade in Algeria, Belgium, Finland, India, and the Netherlands. The majority of these cases had no prior exposure to livestock [10], [12], [15]. These results underscore the usefulness of integrating these assays into *S. aureus* CC398 surveillance programs and epidemiological studies.

IEC is present at a high frequency among *S*. *aureus* clones circulating in humans but appears to have been lost during multiple independent human-to-animal host jumps by *S. aureus* belonging to CC398 [3], CC5 [27], CC97 [28], and CC8 [29]. In addition, IEC is absent from a porcine *S*. *aureus* CC398 isolate (P23-14_SD4.1) belonging to the human clade [3]. The independent loss of IEC in multiple *S*. *aureus* lineages provides strong support for the view that IEC is undergoing purifying selection in animal hosts. By contrast, the presence of IEC in a porcine *S*. *aureus* CC398 isolate displaying *spa* type t899 (UB08116) within the livestock clade supports that IEC has been reacquired [3]. In the present study, we also identified IEC in four human LA-MRSA CC398 isolates that were placed in the livestock clade by the canSNP assays, including three isolates displaying *spa* type t899 from Denmark and France and one isolate displaying *spa* type t034 from Denmark. Of note, a recent study provided support for the view that acquisition of IEC has facilitated animal-to-human host jumps by livestock-associated *S*. *aureus* CC97 isolates, leading to community spread worldwide [28]. It is therefore possible that reacquisition of IEC enables livestock-associated *S*. *aureus* CC398 to spread in human populations in a livestock-independent manner. We are currently monitoring for livestock-associated *S*. *aureus* CC398 harboring IEC in persons with and without livestock exposure to assess the risk for the emergence of a sustainable community reservoir for livestock-associated *S*. *aureus* CC398.

Based on the presence/absence pattern of *tet*(M), it is tempting to speculate that the tetracycline resistance phenotype can be used as a marker for *S*. *aureus* CC398 isolates belonging to the livestock clade. However, all *S*. *aureus* CC398 isolates from Finland that were placed in the human clade by use of the canSNP assays (n* = *5) were resistant to tetracycline [12]; these isolates were subsequently shown to carry the tetracycline resistance gene *tet*(K) rather than the *tet*(M) gene (unpublished data). Thus, whereas tetracycline susceptibility may have utility as a screening tool to exclude *S*. *aureus* CC398 isolates belonging to the livestock clade, the tetracycline resistance phenotype seems to be less useful for exclusion of isolates belonging to the human clade.

In conclusion, the present study has underscored the usefulness of two molecular genotyping methods for defining the major host-associated *S*. *aureus* CC398 clades and has illustrated the power of integrating surveillance, molecular epidemiology, bioinformatics, and microbiology. Results from the two methods can be used independently for epidemiological investigations and source tracking and can be combined to screen for evolutionary signs of adaptation to new hosts and to predict public health risk. Integrating the assays into surveillance programs will aid in determining which reservoirs and bacterial factors are responsible for the increasing prevalence of *S*. *aureus* CC398 in the community.

## Supporting Information

Table S1 **Molecular characteristics of 272 human** ***Staphylococcus aureus*** **CC398 isolates.**
(XLSX)
